# Low-energy Resistive Random Access Memory Devices with No Need for a Compliance Current

**DOI:** 10.1038/srep10409

**Published:** 2015-05-18

**Authors:** Zedong Xu, Lina Yu, Yong Wu, Chang Dong, Ning Deng, Xiaoguang Xu, J. Miao, Yong Jiang

**Affiliations:** 1 State Key Laboratory for Advanced Metals and Materials, School of Materials Science and Engineering, University of Science and Technology Beijing, Beijing 100083, China; 2 Institute of Microelectronics, Tsinghua University, Beijing 100084, China

## Abstract

A novel resistive random access memory device is designed with SrTiO_3_/ La_2/3_Sr_1/3_MnO_3_ (LSMO)/MgAl_2_O_4_ (MAO)/Cu structure, in which metallic epitaxial LSMO is employed as the bottom electrode rather than traditional metal materials. In this device, the critical external compliance current is no longer necessary due to the high self-resistance of LSMO. The LMSO bottom electrode can act as a series resistor to offer a compliance current during the set process. Besides, the device also has excellent switching features which are originated in the formation of Cu filaments under external voltage. Therefore it provides the possibility of reducing power consumption and accelerating the commercialization of resistive switching devices.

The requirements for computing technologies continuously rise due to the unprecedented computing performance we ever want. The resistive switching devices with a simple metal/insulator/metal structure have been extensively studied as promising candidates for the non-volatile memory or logic applications due to their simple structure, excellent scalability, fast switching speed, high endurance, low energy consumption and cost^1^,^2^,^3^,^4^. The resistive memory cell sandwiched between two vertically aligned electrodes constitutes crossbar memory arrays in which the building blocks, referred to as bit cells, possess two or multiple stable resistance states^5^,^6^.

On the way to commercialization of the resistive switching devices, some key challenges are still needed to be solved^7^,^8^. For many devices, the resistive switching phenomenon is believed to be caused by the formation and rupture of conducting filaments^1^,^9^. For the traditional metal/insulator/metal structure, it is necessary to apply a compliance current to control the scale of filaments and a large erasing current to reset the device, which causes high energy consumption. Recently, the p^++^ Si/a-Si/Ag devices were studied that did not require a current limit in the external circuit during programming due to an intrinsic series resistance^10^,^11^. However, the doped polycrystalline silicon as the electrodes in oxide switching devices brings unavoidable oxidation of the electrodes, which maybe affects the device performance. Conductive oxides are very small parts of the oxide family. Most of their resistivities are as low as metal and cannot be well controlled (poor freedom). In this paper, the conductive 3d transition metal oxide La_2/3_Sr_1/3_MnO_3_ (LSMO) is employed as the bottom electrode replacing traditional metal electrodes, such as Au and Pt. For 3d complex perovskite oxides, the emergence of exotic electronic phases from intricate coupling of charge, spin, orbital and lattice degrees of freedom has been a central theme of research in modern condensed matter physics^12^. The perovskite manganites exhibit a remarkably rich variety of structural, magnetic, and transport properties. They have been widely used for the electrodes of ferroelectric tunnel junctions (FTJs), magnetic tunnel junctions (MTJs) and other ferroelectric or multiferroic devices^13^,^14^,^15^. These properties make them have more freedom than simpler materials and can bring more features for resistive switching, for example, magnetism^16^. And their resistance can be controlled in a very wide range by various ways: adjusting ionic radii, doping, magnetic field, electric field, temperature, pressure and photoexcitation^17^. The LSMO bottom electrode possesses high self-resistance and can act as a series resistor to offer a compliance current, which could simplify the structure of devices. Similarly, the reset process can be realized under low current, which reduces the energy consumption of the devices.

## Results

**Figure 1:**
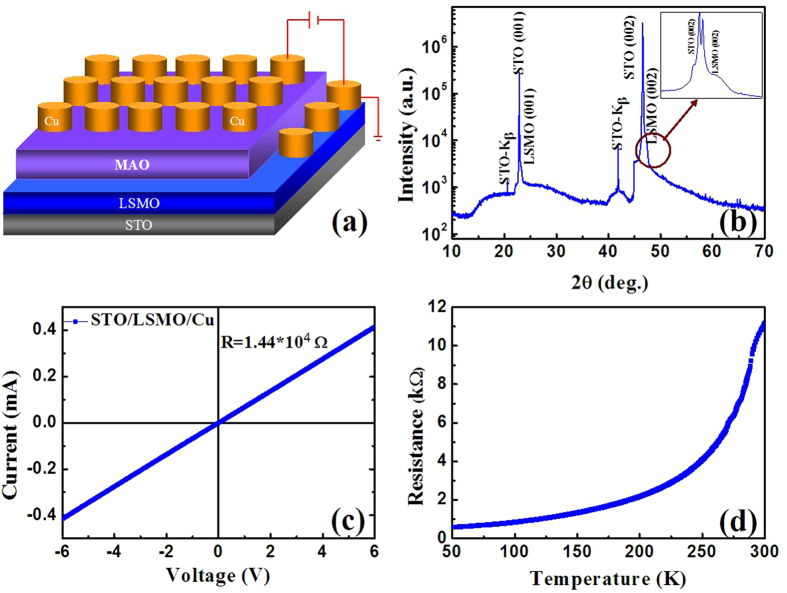
(**a**) Schematic diagram of the sample layout and measurement configuration. (**b**)The XRD patterns of the STO/LSMO/MAO/Cu device. (**c**) The I−V characteristics of the STO/LSMO/Cu film. (**d**) The temperature dependence of the resistance for the STO/LSMO /Cu film.

Under relatively small compressive or tensile strain, the LSMO thin film shows metallic behavior with slightly reduced Curie temperature, while it becomes semiconductive or insulating if a large compressive or tensile strain is applied^18^. The metallic epitaxial LSMO film on the STO substrate is selected as the bottom electrode in our device. MgAl_2_O_4_ (MAO) is a good insulator under only 10 nm thickness that has high initial resistance and low leakage to bring huge R_off_/R_on_ value. Therefore, MAO is a good choice for the switching layer. Fig. 1a shows a schematic diagram of the sample layout and the measurement configuration. The positive bias voltage is applied to the top Cu electrodes, and the bottom electrode LSMO is connected to ground through the Cu electrode on the LSMO layer. The structure of the film is determined by X-ray diffraction (XRD), as shown in Fig. 1b. Only (00l) peaks of LSMO and the STO substrate are observed, indicating the LSMO film is epitaxially grown on STO with a pure phase. A broad halo peak is observed without any peak related to the crystalline phase of MAO, indicating the MAO layer is still amorphous even after 500 °C annealing. The conductive feature of the LSMO layer with the Cu electrodes is typical Ohmic conduction and the self-resistance is 14.4 kΩ, as shown in Fig.1c. The resistance-temperature (R-T) curve indicates that the LSMO is a metallic conductor, as shown in Fig. 1d.

**Figure 2:**
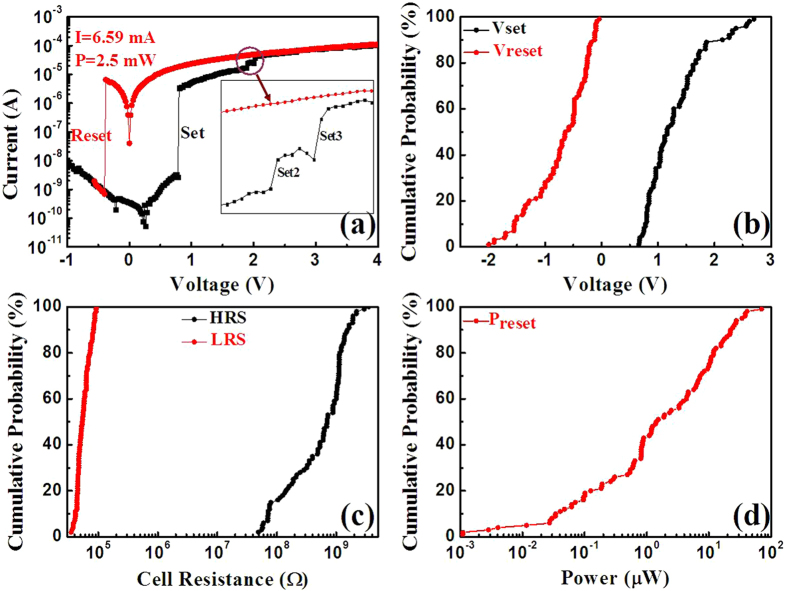
(**a**) The I−V characteristics of the resistive switching for the STO/LSMO/MAO/Cu device. (**b**), (**c**) and (**d**) show the cumulative probability of the set and reset voltages, the resistance for both HRS and LRS under a read bias of 0.5 V and the reset power, respectively.

Figure 2a depicts typical bipolar resistive switching I–V curves for the sample on a semilogarithmic scale (first curve), and no obvious forming process is observed, which implies that the device actually shows a forming-free BRS behavior, which is beneficial to the practical application, similar as the Ag/SiO_2_/ITO device^19^. A negative voltage sweep even up to 10 V and cannot change the resistance state of an as-fabricated device, not shown here. The device’s resistance abruptly changes from a high resistance state (HRS) to a low resistance state (LRS) while a positive bias voltage increases from 0 to 0.78 V without a compliance current, named as the set process. When the voltage is subsequently increased to 4 V, the device still maintains the switching characteristics without any damage. When a negative bias voltage of -0.38 V is applied, the resistance turns back to HRS, called the reset process. The reset current is 6.59 μA, and the power obtained is about 2.5 μW which is pretty low. A near 10^4^ memory window is produced in this device.

Successive 100 switching cycles are carried out on the device, and the statistical analysis is made to get further information about the switching stability, as shown in Fig. 2b-d. The memory device exhibits excellent stability in term of its resistive switching behavior. The distribution of both set voltage (V_Set_, from 0.66 to 2.7 V) and reset voltage (V_Reset_, from -2.0 to -0.04 V) are narrower than those of traditional Metal/electrolyte/Cu devices^20^,^21^. Furthermore, the coefficient of variation (standard deviation/mean value) is 0.38 and 0.70 for V_Set_ and V_Reset_ obtained, respectively, which are lower than reported values^22^,^23^. For the resistance of HRS and LRS, the former exhibits a broad range of dispersions at V_read_ = 0.5 V, while the later presents better uniformity. The reset power also has a broad range of dispersions which mainly concentrates on 0.1 to 10 μW and the maximum value is 70 μW.

**Figure 3:**
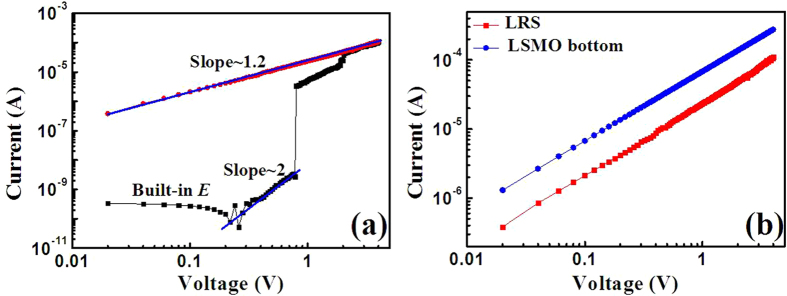
(**a**) The fitting results of the HRS and LRS for the I−V curves in positive voltage region for the STO/LSMO/MAO/Cu device. (**b**) The conductive behavior of the device’s LRS and the LSMO bottom electrode in positive voltage region.

To further understand the conduction and switching behaviors of the device, the I-V curve in positive voltage region is fitted for both HRS and LRS in double logarithmic coordinate, as shown in Fig. 3a. It is obvious that the I-V curve can be divided into two completely different sections (0 to 0.22 V and 0.22 V to Vset) for the HRS. The current decreases with the increase of bias voltage from 0 to 0.22 V, which means the existence of a reverse built-in electric field. A reverse built-in electric field for the low voltage region almost appears in each cycle, not shown here. Then, the current increases with the increase of bias voltage from 0.22 V to V_set_, and the charge transport behavior is in good agreement with the space-charge-limited conduction (SCLC)^24^. For the LRS, I-V curve shows an Ohmic conduction behavior. The completely different conduction behaviors between the LRS and HRS suggest that the filament effect rather than tunnel one dominate the switching process^25^. As shown in Fig. 3b, the conductive behaviors of the LRS and the LSMO bottom electrode are quite similar, which proves the electrons are injected from Cu into LSMO layer rather than sub-oxide or tunneling effect. Y. C. Yang, *et al.* demonstrated that the metal Cu could be easily oxidized, migrated and reduced in oxide electrolyte under enough large voltage for the cation-migration-based electrochemical metallization memory (ECM) devices^26^. From the inset of Fig. 2a, more than one distinguishable current step is observed in the I-V curve, similar to the reports from M. Liu and co-works^27^,^28^, which reveals the successive growth of multiple Cu filaments in the LRS state. Therefore, the resistive switching is originated in the formation of Cu filaments under external bias voltage for the STO/LSMO/MAO/Cu device. Under a reverse bias voltage, the filaments could be broken by the anodic dissolution of Cu according to the reaction: Cu→Cu^2+^+2e^−^. For our ECM device, the built-in electric field phenomenon is observed. I. Valov, *et al.* proved that the built-in electric field originated from nanobatteries in redox-based resistive switches^29^. And S. Gao, *et al.* demonstrated that the HRS of Cu/P3HT:PCBM/ITO raised an obvious built-in electric field under light, which was originated from electrons and holes generated by light in both state^21^. So we infer that the electrons are gathered at the LSMO/MAO interface forming a negative built-in electric field with the holes in LSMO.

**Figure 4:**
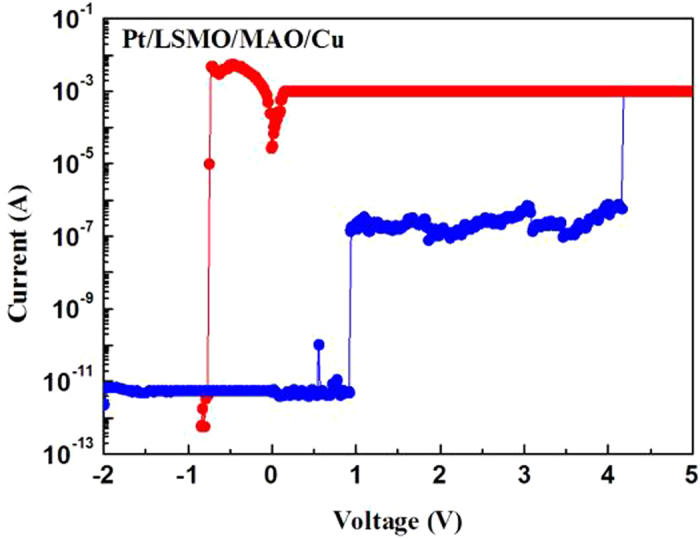
The I−V characteristics of the resistive switching for the Pt/LSMO/MAO/Cu device.

Furthermore, we have prepared a Pt/LSMO/MAO/Cu device. The switching characteristic is shown in Fig. 4. The Pt/LSMO/MAO/Cu device presents bipolar switching behavior, and the extra compliance current is essential to realize the reproducibility of resistance switching. The LRS is far less than the resistance of LSMO, which proves the Cu filament can grow in the MAO layer when the LSMO works as a conductive buffer layer. The two current steps are obvious. It is because the Cu filament firstly grows in the MAO layer and then the Cu is electrochemical metalized in the polycrystalline LSMO layer.

**Figure 5:**
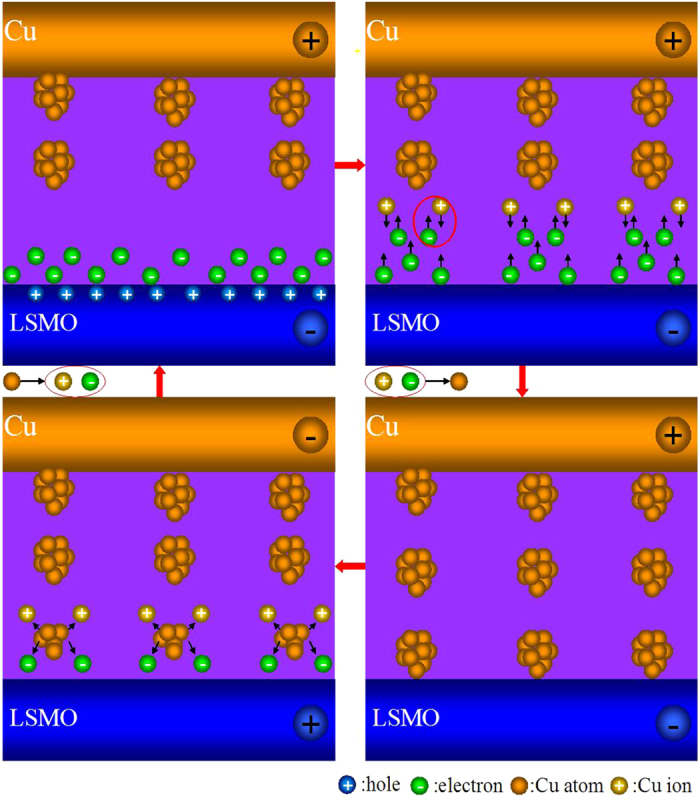
Schematic diagram of the filament resistive switching: (**a**) the built-in field, (**b**) the SCLC conduction process, (**c**) the set process and (**d**) the reset process.

Based on the above analysis, the detailed mechanism of the filamentary resistive switching is proposed as the below four processes, as shown in Fig. 5. At the beginning, an external voltage overcomes the built-in electric field and the electrons compensate the holes in LSMO. Then, the electrons are locally trapped by Cu discrete nanoclusters (residual filaments) in the MAO layer^4^,^26^,^30^. Afterwards, the filaments initiated from the interface of MAO/Cu grow towards the inert electrode as discrete nanoclusters, according to the cathodic deposition reaction: Cu^2+^+2e^−^→Cu. Finally, the Cu filaments are dissolved by a reverse electric field and the device comes back HRS according to the reaction: Cu→Cu^2+^+2e^−^.

The compliance current for the ECM resistive switching devices is critical. The LSMO bottom electrode possesses high self-resistance and can act as a series resistor to offer a compliance current to control the scale of Cu filaments which determines the reset current and power. The consumption of the STO/LSMO/MAO/Cu device can be effectively reduced by the application of LSMO bottom electrode, no longer need of an extra resistor.

## Conclusion

We have studied the effect of epitaxial LSMO bottom electrode on the resistance switching of the STO/LSMO/MAO/Cu device. The resistance can be switched with no need for a compliance current because the LSMO electrode possesses high self-resistance to act as a series resistor. Furthermore, a much less reset power is achieved in the switching device with the LSMO bottom electrode. The origin of the resistance switching is demonstrated to be the formation of Cu filaments under external voltage.

## Methods

A 5-nm-thick LSMO film was in situ grown by pulsed laser deposition (PLD) on a SrTiO_3_ (STO) (001) single crystal substrate at 750 °C and 0.1 mbar partial pressure of oxygen. A KrF excimer laser (λ = 248 nm) with a laser fluence of ~1 J/cm^2^ was used for ablating the high purity LSMO target (99.99%). After the growth, the sample was slowly cooled to room temperature in vacuum at a rate of 5 °C/min. A 10-nm-thick MgAl_2_O_4_ (MAO) memory layer was deposited on the LSMO bottom electrode by rf-magnetron sputtering at room temperature with the power of 60 W. Then, the thin film was annealed under O_2_ atmosphere for 10 minutes at 500 °C to reduce oxygen vacancies and induce the crystallization of MAO. A 200-nm-thick Cu top electrode was then deposited on the oxide heterostructure by dc-magnetron sputtering at room temperature with a shadow mask (the diameter = 200 μm). The R-T curve of STO/LSMO/Cu was measured by a Quantum Design Physical Properties Measurement System (PPMS). The switching characteristics were investigated using a Keithley 4200 semiconductor characterization system.

## Author Contributions

Z.D.X. conceived and designed the study, and carried out the sample preparation and testing. L.N.Y and C.D. assisted to process the data. Prof. X.G.X. and Prof. Y.J. planned and supervised the study. Z.D.X. wrote the paper and L.N.Y, Prof. X.G.X. and Prof. Y.J. gave out the amendments for manuscript. All authors contributed to the scientific discussions, including Y.W., J.M. and N.D.

## Additional Information

**How to cite this article**: Xu, Z. *et al.* Low-energy Resistive Random Access Memory Devices with No Need for a Compliance Current. *Sci. Rep.* **5**, 10409; doi: 10.1038/srep10409 (2015).
